# Persistence associated with extractive foraging explains variation in innovation in Darwin’s finches

**DOI:** 10.1093/beheco/arad090

**Published:** 2023-10-28

**Authors:** Paula Ibáñez de Aldecoa, Sabine Tebbich, Andrea S Griffin

**Affiliations:** Department of Behavioural and Cognitive Biology, Biologiezentrum University of Vienna, Djerasiplatz 1, 1030 Vienna, Austria; Department of Behavioural and Cognitive Biology, Biologiezentrum University of Vienna, Djerasiplatz 1, 1030 Vienna, Austria; School of Environmental and Life Sciences, University of Newcastle, University Drive, Callaghan, NSW 2308, Australia

**Keywords:** Avian cognition, foraging ecology, innovativeness, interspecies comparison, multi-access box, problem-solving

## Abstract

The capacity to create new behaviors is influenced by environmental factors such as foraging ecology, which can lead to phylogenetic variation in innovativeness. Alternatively, these differences may arise due to the selection of the underlying mechanisms, collaterally affecting innovativeness. To understand the evolutionary pathways that might enhance innovativeness, we examined the role of diet breadth and degree of extractive foraging, as well as a range of intervening cognitive and behavioral mechanisms (neophilia, neophobia, flexibility, motivation, and persistence). Darwin’s finches are very suitable to this purpose: the clade is composed of closely related species that vary in their feeding habits and capacity to develop food innovations. Using a multi-access box, we conducted an interspecies comparison on innovative problem-solving between two diet specialists, extractive foragers (woodpecker and cactus finch), and two diet generalists, non-extractive foragers (small and medium ground finch). We predicted that if extractive foraging was associated with high innovativeness, variation would be best explained by species differences in persistence and motivation, whereas if diet generalism was the main driver, then variation would be due to differences in flexibility and responses to novelty. We found a faster capacity to innovate and a higher persistence for extractive foragers, suggesting that persistence might be adaptive to extractive foraging and only secondarily to innovation. Our findings also show that diet generalism and some variables linking it to innovation were unrelated to innovativeness and call for the development of joint experimental approaches that capture the diversity of factors giving rise to novel behaviors.

## INTRODUCTION

Innovation can be defined as a compendium of mechanisms that give rise to new behavioral interactions between an agent and its social or physical environment, resulting in the introduction of novel behavioral variants into the population’s repertoire (Reader and Laland 2003). Innovations can be highly advantageous since they allow organisms to potentiate their resilience and adjust to changing environments by increasing the range of exploitable resources (Griffin and Guez 2014; Reader et al. 2016; Ducatez et al. 2020). It is now well established that some phylogenetic clades are significantly more innovative than others, and much research effort has been invested in understanding why. For example, comparative analyses on innovation rates conducted in a world-wide sample of over 700 avian species (Ducatez et al. 2015) showed that diet generalists have higher numbers of technical innovations (i.e., novel foraging techniques) and larger brains (Overington et al. 2009; Ducatez et al. 2020). This pattern has been interpreted to indicate that diet generalism enhances cognition, which in turn mediates technical innovative capacity (Sol 2003; Ducatez et al. 2015). Therefore, specific foraging ecologies, including diet generalism, may lead directly to increases in the capacity to innovate (henceforth referred to as innovativeness). However, this is not the only possible evolutionary pathway for generating phylogenetic variation in innovativeness. Another possibility is that specific ecological conditions favor one (or many of the) behavioral mechanisms that underlie innovation, with only collateral (indirect) effects on innovation capacity (Griffin and Guez 2016).

The process of innovation includes several stages, together with the mechanisms acting on them (Tebbich et al. 2016). The first stage of innovation involves approaching a novel situation (e.g., a novel stimulus that may be food), which depends on an individual’s neophilic or neophobic predisposition. These traits are driven by the cost/benefit ratio of approaching or avoiding novelty and, consequently, influence the probability of innovating (Thorpe 1956; Greenberg and Mettke-Hofmann 2001; Mettke-Hofmann et al. 2002; Mettke-Hofmann 2014). Neophobia is thought to be advantageous in environments where predation risk is high (Greenberg and Mettke-Hofmann 2001). In complex environments, the benefits of approaching unfamiliar objects (neophilia) are considered high and may be greater for animals with a generalist lifestyle (Greenberg 1990; Greenberg and Mettke-Hofmann 2001; Mettke-Hofmann 2014). For example, Tebbich et al. (2009) found that among 13 species of Darwin’s finches, those with a greater diet diversity were also more willing to explore novel objects. A similar relationship between generalism and exploration has been found in baboons (*Papio ursinus*) and geladas (*Theropithecus gelada*; Bergman and Kitchen 2008) as well as in satyrine butterflies (*Lopinga achine* and *Pararge aegeria*; Leimar et al. 2003). One idea is that generalists encounter a greater range of novel stimuli during development, leading them to maintain a lower avoidance of novelty later in life (Greenberg 1990). Regardless of the mechanism, it is likely that foraging ecologies that modify animals’ responses to novelty influence the evolution of innovativeness indirectly.

The second step of innovation involves active engagement with the novel opportunity (Tebbich et al. 2016). Motivation to contact the affordances of an opportunity along with persistent engagement with the opportunity is known to increase the chances of discovering a way to exploit it (Massen et al. 2013; Griffin and Guez, 2014; Guez and Griffin, 2016; van Horik and Madden 2016). Foraging modes that involve extracting hard-to-access food items are characterized by high levels of motivation and persistence, which are likely to increase innovative potential (Biondi et al. 2021). Finally, the third step of innovation involves the capacity to shift from an established preference if an ongoing behavior is not successful (Tebbich et al. 2016), that is, flexibility (Sol et al. 2002, 2005). While diet generalists feed on a large range of food types that require flexibility to apply different manipulation techniques, extractive foraging, with its high levels of persistence, is associated with a low sensitivity to absence of reinforcement (Tebbich et al. 2010, 2011; Teschke et al. 2011; Diquelou et al. 2016). Therefore, specific foraging modes may lead indirectly to increases or decreases in innovativeness depending on which innovation mechanisms they potentiate.

To investigate how innovativeness evolves, we need a phylogenetically kindred, highly innovative, yet ecologically diversified taxon. Although numerous efforts have been put into investigating the aforementioned factors, there is a research gap with respect to comparisons between closely related, ecologically diverse species. Darwin’s finches are a unique system on which to undertake this type of study: they constitute one of the most innovative avian clades (Grant 1986; Tebbich et al. 2004, 2010), with several species incorporating atypical nutritive resources and adopting unusual techniques to exploit them. Some of these examples include using twigs or cactus spines to extract arthropods embedded in cavities (woodpecker finch; Eibl-Eibesfeldt 1961), plucking ticks and other ectoparasites from reptiles, or feeding on sea lions’ placenta (both behaviors reported on small and medium ground finch; MacFarland and Reeder 1974; Grant and Grant 2008). The taxon is composed of 17 closely related species (Farrington et al. 2014) that mainly differ morphologically in their beak shape (Grant and Grant 1994) and associated foraging ecology (i.e., diet breath or spectrum of foods consumed and strategies to gain access to food resources; Grant 1986; Schluter 2000). Despite their genetic relatedness, these passerines are subjected to different selective pressures and interact with relevant resources differently (Grant 1986). Hence, they constitute a very suitable model to perform interspecies comparisons on the role of foraging ecology on the mechanisms that facilitate innovativeness (Tebbich and Teschke 2014).

In this study, we investigated (1) whether Darwin’s finches species who differ in their diet breadth and dependency on extractive foraging also differ in their capacity to innovate and (2) which behavioral mechanism(s) best explain these differences. To that end, we compared innovativeness of two species that can be considered as diet specialist, extractive foragers (woodpecker finch, *Camarhynchus pallidus*; and cactus finch, *Geospiza scandens*) versus two species that have predominantly a generalist, non-extractive diet (small ground finch, *Geospiza fuliginosa*; and medium ground finch, *Geospiza fortis*), rich in various seeds and fruits as well as occasionally arthropods during chick rearing. While woodpecker finches are mainly insectivorous and retrieve a high percentage of embedded food by probing and pecking (Tebbich et al. 2004), cactus finches are *Opuntia* specialists that exploit flowers, nectar, and seeds, but also insects from rotting cactus pads that they retrieve by pecking (Grant and Grant 1981). We used a set of standardized cognitive and behavioral paradigms, including a Multi-Access Box (MAB), to measure interspecies variation in innovativeness and relate it to variation in innovation mechanisms (Auersperg et al. 2011; Biondi et al. 2021; Jacobson et al. 2022; Petelle et al. 2023). Our MAB featured a battery of four tasks presented simultaneously, leading to the same, enclosed reward, and allowing for discovered solutions to be blocked. The MAB allowed us to measure not only innovativeness but also flexibility, motivation, and persistence. Additionally, we presented two separate tasks to assess neophobic and neophilic responses.

Here, we used the same approach as Diquelou et al. (2016), who measured innovativeness and its mechanisms among a community of urban native and invasive birds in Australia. We first tested whether species with known differences in diet generalism and foraging mode differed in innovativeness. Then, we identified which mechanisms explained this variation. Finally, we tested whether species differed in those mechanisms. We predicted that innovativeness would vary among diet generalists (small and medium ground finches) and extractive foragers (cactus and woodpecker finches) so that if extractive foraging was associated with high innovativeness, then it would be best explained by species differences in persistence and motivation. If, on the contrary, diet generalism was associated with high innovativeness, then it would be mainly due to species differences in flexibility and responses to novelty.

## MATERIALS AND METHODS

### Ethics approval

Permission to conduct this study was granted by the Galápagos National Park and the Charles Darwin Research Station (Project PC-93-19). Housing conditions complied with the Austrian Federal Act on the Protection of Animals (Animal Protection Act –§ 24 Abs. 1 Z 1 and 2; § 25 Abs. 3—TSchG, BGBl. I Nr. 118/2004 Art. 2). As all experiments were appetitive, noninvasive, and based exclusively on behavioral tests, they were not classified as animal experiments under the Austrian Animal Experiments Act (§ 2. Federal Law Gazette No. 501/1989). All birds were held for the minimum amount of time required to complete the experiments and then released at their site of capture.

### Subjects, housing conditions, and diet

Thirty-nine wild Darwin’s finches of the following species participated in this study (number of individuals of each species between brackets): small ground finch (9), medium ground finch (11), cactus finch (10), and woodpecker finch (9). To catch the birds, we used mist nets (716/6 Ecotone®) and a pocket speaker with a playback call to attract them; catching sites were situated at least 1 km away from the nearest urban area. Upon capture, we weighed each individual to the nearest 0.1 g using an electronic scale and banded them with an identificatory metal ring on the leg. Then, we placed birds singly for around 48 h in a wooden habituation cage (dimensions: 1 × 0.5 × 0.5 m) to acclimate them to captivity. After habituation, subjects were housed individually in outdoor aviaries (dimensions: 1 × 2 × 2 m), visually (but not acoustically) isolated from each other by opaque white fabric that covered the aviary walls. One of the walls had a small opening that served as a window to observe the bird from the experimental compartment during testing sessions, after which the window was closed again. In all aviaries, at least one of the walls directly faced the surrounding natural environment and remained uncovered with fabric. Each aviary was furnished with anti-mosquito mesh and natural tree branches serving as perches on the walls, as well as gravel and a small bathing pool on the floor for enrichment. A concrete block with a tabletop was located near one of the walls to place the main feeding dish; during the testing sessions, food was removed, and the experimental apparatus was placed on the tabletop instead.

All subjects received a diet consisting of a mixture of flax seeds, raw quinoa, rehydrated raisins, hardboiled egg and crushed eggshell, finely ground walnuts, grated yuca and carrot, fresh sliced fruits, and dry insect pâté (Versele-Laga Orlux®), as well as ad libitum drinking water supplemented with 5% Baycox (Bayer®) to treat against coccidiosis (dosage: 3 mL per liter of water). Food was presented in shallow plastic dishes on the tabletop as well as in hopper feeders hanging from the walls. In addition, as a reward for each solved trial, insectivorous birds (cactus and woodpecker finches) received a moth (previously caught with a handmade light trap), while the small and medium ground finches received papaya, different seeds and/or rice. Prior to each experimental session, birds were food-deprived for 2 h to increase their appetite and motivation to participate. Before testing, every morning, the aviaries were properly cleaned and disinfected by removing the dirty gravel and replacing it with fresh one, scrubbing all surfaces with an ammonium-based disinfectant, and soaking the feeding and water dishes as well as the bathing pool in biodegradable washing liquid. Birds spent an average of two weeks in captivity, after which they were released back to the catching site. Data collection took place between September and December 2019 (the dry season) at the aviaries of the Charles Darwin Research Station in Santa Cruz Island (Galápagos, Ecuador).

### Apparatus and procedures

#### The Multi-Access Box

**Figure 1:**
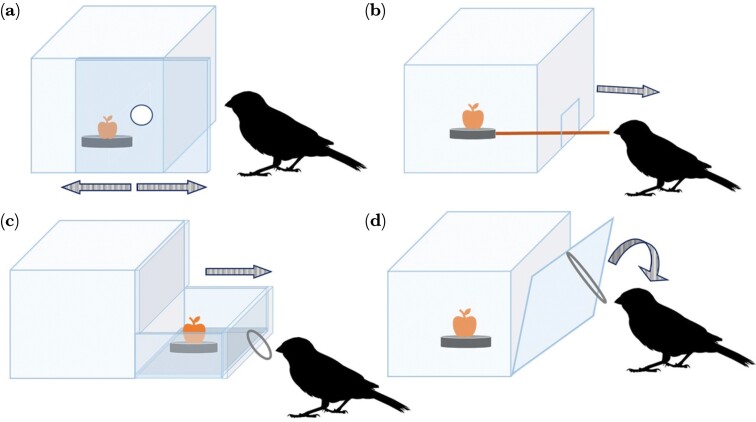
The MAB showing its four mechanisms and the respective features that birds had to interact with to obtain the reward (symbolized by an orange-colored apple), which was placed in a black bottle cap. (a) Sliding door: the interactive feature was a hole placed in the middle of one of the walls; (b) Toothpick: birds had to grab the blunt end of a wooden toothpick (shown in brown) that popped out of a small gate; (c) Drawer: the opening mechanism was a metal ring on the lower half of one of the walls; (d) Trap: birds had to pull a string loop located on the top half of the trap door. Arrows denote the direction of movement displayed by the bird to open each mechanism.

**Figure 2:**
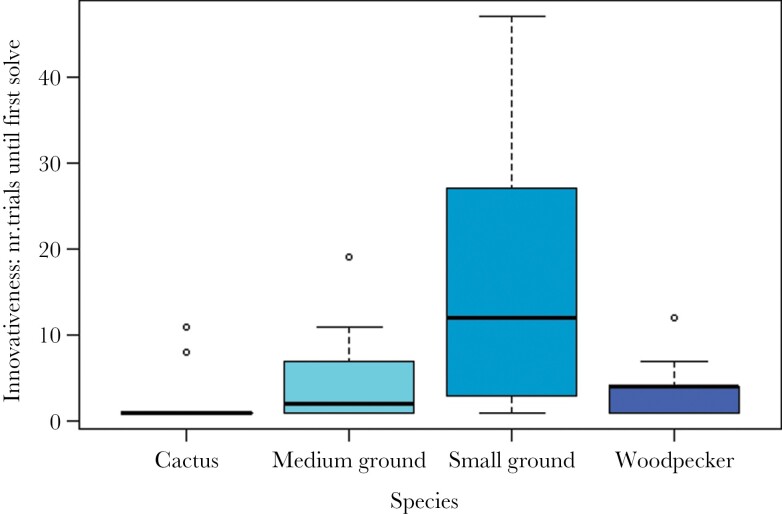
Species differences in innovativeness, measured as the number of trials needed to solve the first mechanism (model 1). Species are depicted in different colors: pale blue (cactus finch), cyan (medium ground finch), slate gray (small ground finch) and navy blue (woodpecker finch). In boxplot, the top and bottom ends of the box are the lower (Q1 = 25%) and upper (Q3 = 75%) quartiles. The box covers the interquartile interval, where 50% of the data is found. The horizontal line that splits the box into two is the median. The upper and lower whiskers represent the maximum and minimum, respectively (without outliers, which are represented by circles).

**Figure 3:**
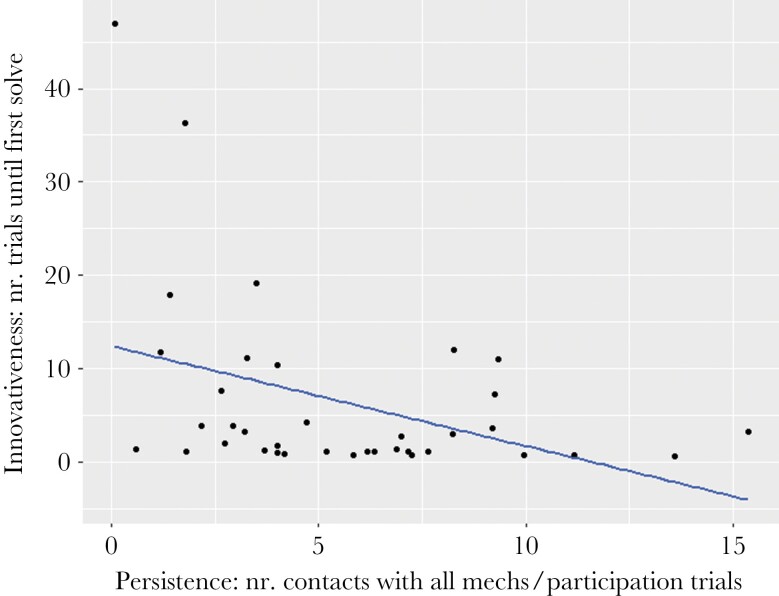
Effect of persistence (number of contacts with all mechanisms per participation trial) on innovativeness (number of trials needed until first solve; model 2). Plot based on raw data; values correspond to all species clustered together.

**Figure 4:**
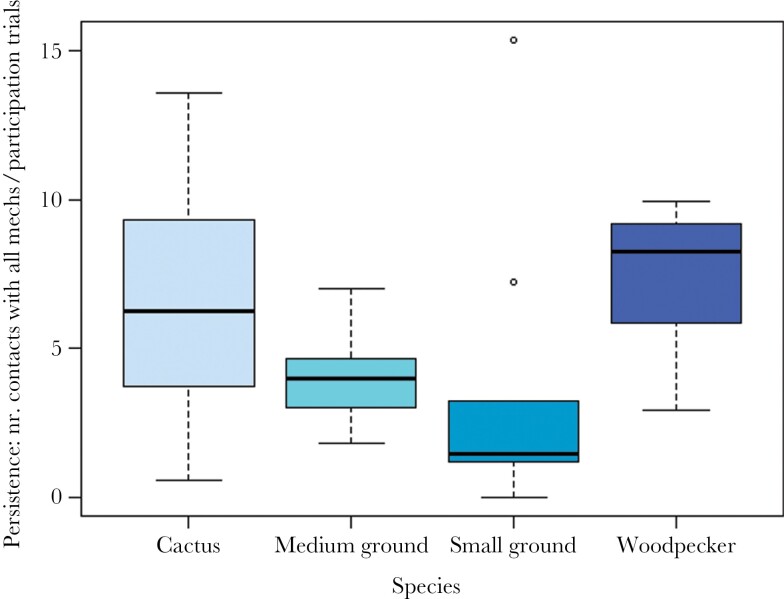
Species differences in persistence, measured as the number of contacts with all mechanisms per participation trial (model 3).

Our testing apparatus, the MAB, was a 7 cm^3^ translucent Plexiglas cube box with four alternative opening mechanisms, each of them situated on one of the side walls. The goal for the subject was to interact with the box and open it via one of the mechanisms available, retrieving the food reward contained in a black bottle cap placed in the center of the box’s floor, which was visible from the outside yet unreachable unless the bird solved the task. Each opening mechanism displayed a feature or element that the bird had to engage with to unblock it and successfully open the box: (1) Sliding door: a 7 mm diameter hole placed in the middle of one of the walls allowed it to be slid leftwards or rightwards with the tip of the beak or the claws to open the door and gain access to the reward (Figure 1a); (2) Toothpick: a wooden toothpick, with one end connected to the bottle cap containing the reward inside the MAB and the other blunt end popping out of a small gate on the MAB, which the bird had to lightly pull out to drag the reward toward itself (Figure 1b); (3) Drawer: a metal ring located on the lower half of one of the walls, which the bird had to pull to open the drawer and collect the reward (Figure 1c); (4) Trap: a string loop located on the top half of a trap door, which the bird had to pull in a 45° angle to open. In the inner side of the MAB, the trap had a hidden plastic hinge on the bottom half and a small magnet on top to keep it closed unless the mechanism was operated by the bird (Figure 1d).

#### Testing sessions

Prior to formal testing and immediately following acclimatation to captivity, habituation to the MAB commenced. For this, the feeding dishes were removed, and the MAB with all its walls open and containing a bottle cap with food was left on the tabletop while the experimenter observed from the adjacent compartment through the window opened on the fabric covering the wall. Each habituation session lasted 25 min, and birds had to approach the MAB and feed from it six times across different habituation sessions. Once habituation concluded, we proceeded with testing, where a maximum of 14 sessions of ten 5-min trials were given. At the beginning of a testing trial, the MAB (baited outside of the bird’s sight) was placed on the tabletop while the experimenter observed the bird’s performance from the adjacent compartment. After each trial, the MAB was rotated clockwise to change its orientation and control for laterality preferences; in the very first trial, we always started with the sliding door facing the experimenter, and we continued with the toothpick, the trap, and the drawer, keeping this order consistent for all birds. If the bird solved any of the mechanisms within the time allocated, the trial was terminated, the MAB was rebaited and placed back in the aviary proceeding with successive trials until the session was over. If the bird did not touch the MAB during a trial, we conducted a so-called “re-habituation” (rehab) trial, consisting of leaving all mechanisms open for 5 min so the bird could approach and feed from the MAB without solving a mechanism, as done in the habituation phase. A maximum of two rehab trials were administered per session. If the bird did not contact the MAB and eat during a rehab trial, the session was terminated; otherwise, testing was resumed where it was left off. “Participation trials” were those on which at least one contact was made with the MAB (i.e., hence excluding rehab trials and trials where the bird did not touch any mechanism). To block a mechanism (i.e., make it unavailable), a bird had to solve it six times, not necessarily in consecutive trials. The respective mechanism was made unopenable by blocking its interactive feature: in the toothpick door, the protruding toothpick was removed, and the small gate was obstructed with transparent tape from the inside; in the drawer, the ring was removed; in the trap, the string was detached; and in the sliding door, the moving panel was affixed to the sides to make it immobile while the hole was sealed with tape. Blocking of previously mastered mechanisms was intended to stimulate birds to explore the still available mechanisms and, potentially, lead to eventually discovering new opening techniques. At the end of each session, the feeding dish with food was given back, and the window opening in the fabric wall was closed again. All trials were videotaped for further coding using a camcorder attached to a tripod.

#### Variables measured

To perform the interspecies comparison, we included a set of metrics which were operationalized as follows (a detailed description of all measures is shown on Table 1). First, considering that a consequence of innovating is gaining access to novel opportunities, innovativeness was assessed as the latency to discover a novel opportunity (i.e., the number of trials needed until the first mechanism is solved). Then, once a mechanism is blocked, a flexible individual would be able to switch to another one; this switching capacity would allow it to discover more mechanisms within the limited number of sessions provided (i.e., the number of mechanisms discovered reflects flexibility). Next, persistence was assessed as the degree of engagement with the functional components of the task, in our case, the number of contacts with all mechanisms divided by the number of participation trials. Motivation was calculated as the number of rehab trials; in doing so, we aimed to estimate the extent to which a presumably demotivated individual had to be re-motivated after not contacting the task during a trial.

**Table 1:** Variables coded from video footage

Name of variable	Definition	Potential range of values
Flexibility	Number of mechanisms discovered across testing	0–4
Innovativeness	Number of trials needed until first mechanism is solved	1–140 If no trials were solved: NA
Motivation	Number of rehab trials/ number of participation trials	0–28 (max. 2 rehabs/session)
Neophilia	Latency to land on the experimental table (in seconds)	1–1800 If bird did not land on table: 1801 (ceiling value)
Neophobia	Latency to feed next to a novel object minus control feeding latency (in seconds)	−1800 to 1801 (ceiling value) If bird did not feed: ceiling value minus control latency
Persistence	Number of contacts with all mechanisms/ number of participation trials	0 to ∞

#### Reaction to novelty: neophobia and neophilia tests

In addition, we conducted two further tests to measure responses to novelty, namely neophilia and neophobia, together with a control feeding latency (CFL). We used these measures to study a possible effect on performance with the MAB. Each measure was taken in the early morning (between 7h00 and 8h00), only once and on separate days, once birds had been for at least 5 days in captivity and were presumably habituated, allowing for a duration of 30 min (1800 s) in each case (as in Lermite et al. 2017). The CFL test and the neophobia test were performed before birds received their first meal of the day, while the neophilia test was conducted once they had already eaten. The CFL was always the first of the three measures to be collected; then, for each species, half of the individuals underwent the neophobia test first, and the other half took the neophilia test first. In the CFL test, the feeding dish was placed on the tabletop as usual, and the experimenter measured with a stopwatch the time it took the bird to approach the dish and feed. For the neophobia test, the feeding dish was placed next to a novel object (a plastic black and green robot toy). For the neophilia test, a different novel object (a blue and red plastic brush with the bristles facing the center of the table) was placed on the tabletop, this time without the feeding dish. We measured latency to touch the object (either by pecking it with the beak or by sitting on it). A ceiling value (1801 s = 1 s longer than the test duration) was assigned to the latency score if the bird did not touch the object in the neophilia test. In the neophobia test, if a bird did not feed, it received a score calculated as ceiling value minus control feeding latency.

### Behavioral coding

Behaviors were coded from the video footage using the software Loopy (http://loopb.io, loopbio GmbH, Vienna, Austria) by two independent coders, yielding a high interrater reliability (intraclass correlation coefficient [ICC] = 0.78; *P* < 0.001). We generated a dataset containing one value per individual for each of the following variables: flexibility, innovativeness, motivation, neophilia, neophobia, and persistence.

### Statistical analysis

We tested our hypotheses by adapting the model type to the response variable, using generalized linear models (models 1 and 3) and linear mixed-effects models (model 2; see Supplementary Table S1 for details on the fitted models). All statistical analyses were conducted in R software (version 4.0.3; R Core Team 2020) with a significance threshold set at α = 0.05. We used the “lme4” package (Bates et al. 2015) to fit the models and the “sjPlot” package (Lüdecke 2022) to obtain confidence intervals at 95%. Data visualization was performed using the “ggplot2” package (Wickham, 2016). The normality of the residuals was confirmed using the Shapiro-Wilk normality test (function shapiro.test, “stats” package; R Core Team 2020). Each individual was identified with a unique alphanumeric code. As we were not always able to determine birds’ sex or exact age, and since we did not have specific predictions about the effect of these variables, we disregarded them for our analyses. In all models, the cactus (finch) was considered as the reference value for the variable “species” (set by default by R, in alphabetical order). As in Diquelou et al. (2016), we followed a three-step analysis approach to investigate whether species differences in a set of variables explained species differences in their capacity to innovate. We first tested for species differences in our measure of “innovativeness” (model 1, Poisson glm). Then, we evaluated the effect of five independent predictors (“flexibility,” “motivation,” “neophilia,” “neophobia,” “persistence”) on “innovativeness” (model 2, Poisson glmer). Prior to model fitting, we performed z-transformation of the predictors to ease convergence (Schielzeth 2010), and removed NA cases (i.e., two small ground finches who never discovered any mechanism and did not have a score for “innovativeness”), reducing the sample size to 37 from an original of 39. We also included a random effect of “species” to account for repeated measures. Finally, to determine whether between-species difference in innovativeness was attributable to species differences in innovation predictors, we tested whether species differed in the significant predictors identified in the previous step (model 3, Poisson glm).

## RESULTS

In step 1, we found that, compared to cactus finches (the species set as reference), both non-extractive foragers, namely medium ground finches and small ground finches, needed significantly more trials to attain their first solve (*P* = 0.009; *z* = 5.161; *P* < 0.001; *z* = 8.678, respectively; see Table 2a; Figure 2). In step 2 of our candidate variables to explain differences in innovativeness, we only found support for the effect of persistence: a higher proportion of contacts with all mechanisms per participation trial resulted in a smaller number of trials needed to attain the first success (*P* < 0.001; *z* = −4.444; see Table 2b; see Figure 3). Finally, in step 3, we explored species differences in the variable for which significant differences were detected in step 2, that is, persistence. In line with our prediction, we found that medium and small ground finches had a lower persistence (*P* = 0.010; z = −2.573; *P* = 0.002; z = −3.102, respectively; see Table 2c; see Figure 4). Species mean values for flexibility, innovativeness, motivation, neophilia, neophobia, and persistence, as well as a qualitative description of the performance of all species, are reported in Supplementary Tables S2 and Table S3, respectively.

**Table 2:** Coefficients for fixed effects of (a) model 1, (b) model 2, and (c) model 3. Reference value for “species” in all models is “Cactus.”

Response	Predictors	Est.	SE	*P* value	Low CI	Up CI
(a) Model 1 Innovativeness	Intercept [Cactus]	0.993	0.192	<0.001	0.591	1.348
	Species [Medium ground]	0.616	0.235	0.009*	0.166	1.091
	Species [Small ground]	1.848	0.213	<0.001*	1.448	2.286
	Species [Woodpecker]	0.420	0.253	0.097	−0.072	0.926
(b) Model 2 Innovativeness	Intercept	1.592	0.269	<0.001	1.066	2.119
	Flexibility	−0.013	0.119	0.913	−0.246	0.220
	Motivation	−0.234	0.135	0.083	−0.498	0.030
	Neophilia	0.045	0.075	0.546	−0.101	0.191
	Neophobia	0.083	0.082	0.312	−0.078	0.245
	Persistence	−0.577	0.130	<0.001*	−0.831	−0.322
(c) Model 3 Persistence	Intercept [Cactus]	1.887	0.123	<0.001	1.636	2.119
	Species [Medium ground]	−0.501	0.195	0.010*	−0.888	−0.123
	Species [Small ground]	−0.683	0.220	0.002*	−1.128	−0.261
	Species [Woodpecker]	0.090	0.175	0.606	−0.253	0.433

Est., Estimate; SE, standard error; CI, 95% confidence intervals.

*Significant *P* values at α = 0.05 (shown in bold). Number of observations: *N* = 37 (models 1 and 2), *N* = 39 (model 3).

## DISCUSSION

We sought to determine whether interspecies variation in the capacity to innovate (which we refer to as innovativeness) can be explained by species variation in behaviors associated with specific foraging ecologies in Darwin’s finches. Our findings show that diet specialist, extractive foragers were faster innovators in that they solved the MAB for the first time in significantly fewer trials. Among five behavioral facilitators of innovativeness, we found that persistence (measured as number of task contacts per participation trial) was the only variable that explained variation in innovativeness, being higher in the diet specialist, extractive foragers.

Our results confirm previous work showing that woodpecker finches outperform small tree finches (*Camarhynchus parvulus*) in a box-opening task due to making significantly more contacts with the box (i.e., a higher persistence; Teschke et al. 2011). Extractive foraging is characterized by high levels of persistence (Winkler and Leisler 1999), including long bouts of energetic pecking (Tebbich et al. 2004, 2009: 2010) to obtain hard-to-access food (insects under the bark in the case of woodpecker finches and in pads of *Opuntia* cacti in cactus finches). This foraging mode might, therefore, be associated with differences in persistence, which in turn facilitate innovativeness. This is notorious, as it suggests that high levels of persistence might be adaptive to extractive foraging and only secondarily to innovation. Hence, in some species, innovativeness might evolve as a by-product of underpinning behaviors that are under selection within other ecological contexts rather than via selection on innovation outcomes per se.

Persistence has also been found to be an important determent of innovativeness in previous work on the proximate mechanisms of innovation in single species and in population comparisons, both when innovation has been quantified using experimental assays (Massen et al. 2013; Griffin and Guez 2014; van Horik and Madden 2016) and computational models (Guez and Griffin 2016). For example, Jacobson et al. (2022) found that in elephants (*Elephas maximus*), a higher likelihood of success was associated with a greater persistence (i.e., the proportion of time spent interacting with the MAB) and not with other behavioral traits such as exploratory diversity, motivation or neophilia. In another single species study in raccoons (*Procyon lotor*), persistence was one of the main predictors of innovative problem-solving: subjects that spent more time working on a puzzle box were generally more successful (Daniels et al. 2019). Using a comparison of two populations (rural vs. urban caracara chimango, *Milvago chimango*), Biondi et al. (2021) found that a higher effectiveness of solving attempts in the urban population occurred in conjunction with a higher persistence, which enhanced motor flexibility indirectly. Likewise, Sol et al. (2011) found that common mynas (*Acridotheres tristis*) from urbanized areas had higher task contact frequencies, which explained a higher propensity to innovate. The novelty of our work resides in demonstrating differences in experimentally measured innovativeness and persistence among phylogenetically closely related species and that these differences are linked to foraging ecology, more specifically, to extractive foraging.

Our findings further suggest that neither diet generalism nor variables linking it to innovative behavior (flexibility, reaction to novelty) were at play in the MAB performance of Galápagos finches. There is, however, empirical evidence for such relationship (Dukas 1998; Sol 2003, 2015; Ducatez et al. 2015) and much discussion arguing that animals with a generalist lifestyle are more willing to explore novelty, which could, in turn, increase innovation opportunities (Mettke-Hofmann et al. 2002). Therefore, we could have expected the diet generalist finch species tested here (i.e., small and medium ground finches) to be more innovative. It is important to note that even though individual variation is the substrate upon which Darwinian mechanisms of selection operate, experimental tests of innovative behavior do not always reveal expected individual-level correlations. For example, despite field reports showing a relationship between cognition and innovation in birds and primates (Sayol et al. 2016; Herculano-Houzel 2017; Sol et al. 2022), experimental assays have revealed a broad range of alternative mechanisms of innovation (see Logan et al. 2018 for a review), including the one found here with persistence. To reconcile these discrepant patterns at different scales (e.g., large datasets with thousands of species vs. single-species studies), Griffin and Guez (2016) suggested that generalism may select for cognition but simultaneously for other proximate mechanisms, with the latter rather than the former having collateral proximate effects on innovation. This is like the reasoning applied here, in that we suggest that a behavior (persistence) adaptive to a particular foraging mode (extractive foraging) indirectly improved innovation performance.

Additionally, other measures that have been previously found to increase innovativeness (reviewed by Griffin and Guez 2014, but see also Biondi et al. 2021) were unrelated to innovation performance in our study. The MAB creates multiple opportunities to innovate, but when a successful solution is blocked, the subject must switch away from that technique to explore others. Switching away from a previously rewarding solution depends largely upon inhibitory control (the capacity to withhold an action in the face of a more immediate [or expected] reward), which is considered a measure of cognition (for a review, see Audet and Lefebvre 2017). Here, we attempted to capture this ability to flexibly switch by using the total number of solving techniques discovered and found that birds appeared to not use this particular cognitive capacity to solve the MAB. In addition, we did not find a role of motivation, measured as the ratio of re-habituation trials (rehab; where the task was presented with readily available food following a non-participation trial) to the number of participation trials (in which birds interacted with the task). Many other studies used latency to interact with the task for the first time, or total time spent interacting with a task. A review by Griffin and Guez (2014) did not find a consistent role of motivation across studies, a finding the authors attributed to the disparate ways in which this variable is quantified. More work is needed to determine the best way of measuring this latent variable and its relationship to other innovation mechanisms such as persistence. The challenge of operationalizing different behavioral variables in studies conducted at different scales means that it is likely that different intervening variables are revealed by each of these approaches. We recommend that future studies prioritize both developing new experimental innovation assays and new ways of measuring variation in mechanisms among large numbers of species. Only with this joint approach can we hope to reveal the full spectrum of mechanisms that allow animals to innovate and to understand whether innovation is selected directly or as an emergent property of a larger array of traits, which have themselves evolved to deal with environmental variation (Griffin 2016; Tebbich et al. 2016).

In this study, species differences in persistence and their contribution to variation in innovativeness were attributed to species differences in foraging mode, which was the major differentiating ecological factor. However, another possible explanation is that persistence is related to social and/or sexual behavior mediated by hormones. In particular, testosterone has been shown to increase the persistence of food searching in young and adult chicks (Rogers 1974) as well as in male mice (Archer 1977). In our case, by studying a phylogenetically closely related group and outside the breeding season, we think it is unlikely that hormonal differences played a role in variation in persistence. Nevertheless, we encourage future research to explore whether additional factors, such as species differences in androgens, may influence persistence and ultimately affect the capacity to innovate.

## SUPPLEMENTARY MATERIAL

Supplementary material can be found at http://www.beheco.oxfordjournals.org/

arad087_suppl_Supplementary_Material

## Data Availability

Analyses reported in this article can be reproduced using the data provided by Ibáñez de Aldecoa et al. (2023).
