# Supplementary material for: Persistence associated with extractive foraging explains variation in innovation in Darwin’s finches
Source: Behav Ecol. 2023 Oct 28;35(1):arad090. doi: 10.1093/beheco/arad090 (PMC10773301; doi:10.1093/beheco/arad090)
Supplement: arad087_suppl_Supplementary_Material [file arad087_suppl_supplementary_material.doc]

**SUPPLEMENTARY MATERIALS**

**Table S1. Structure of models fitted**. Legend: glm: generalized linear model; glmer: generalized linear mixed-effects model; (~): separates model response (to the left) from predictors (to the right); ‘z’ indicates that variables were z-transformed to ease model convergence; ‘(1|species)’: indicates that “species” was entered as a random effect; family = poisson’: model was fitted to a Poisson distribution.

| Model | R syntax |
| --- | --- |
| Model 1 | glm (innovativeness ~ species, family = poisson) |
| Model 2 | glmer (innovativeness ~ (z.flexibility + z.persistence + z.motivation + z.neophobia + z.neophilia) + (1\|species), family = poisson) |
| Model 3 | glm (persistence ~ species, family = poisson) |

**Table S2. Species mean (± standard deviation)** **values of innovativeness, flexibility, motivation, neophilia, neophobia and motivation.** Values are indicated in the same units as those for the variables shown in Table 1.

| Variable | Cactus  finch | Medium ground finch | Small ground finch | Woodpecker finch |
| --- | --- | --- | --- | --- |
| Flexibility | 3 ± 1,25 | 2,45 ± 1,21 | 0,88 ± 0,6 | 2,77 ± 0,97 |
| Innovativeness | 2,7 ± 3,65 | 5 ± 5,86 | 17,14 ± 17,95 | 4,11 ± 3,55 |
| Motivation | 0,18 ± 0,19 | 0,34 ± 0,41 | 1,04 ± 1,31 | 0,09 ± 0,11 |
| Neophilia | 145,2 ± 404,08 | 658,55 ± 795,02 | 1176,4 ± 845,5 | 263,88 ± 473 |
| Neophobia | 17,2 ± 39,42 | 286,72 ± 553,63 | 758,11 ± 754,6 | 147 ± 226,57 |
| Persistence | 6,51 ± 4,01 | 4,07 ± 1,71 | 3,53 ± 4,94 | 7,28 ± 2,36 |

**Table S3:** **Qualitative description of performance in (i) Medium ground finches; (ii) Cactus finches; (iii) Small ground finches; (iv) Woodpecker finches.** (a) Number of individuals that discovered (solved at least once) 0, 1, 2, 3, or 4 mechanisms (and percentage of the total amount of individuals of that species); (b) Number of individuals who discovered 0, 1, 2, 3, or 4 mechanisms in session 1; (c) Number of individuals who blocked (solved 6 times) 0, 1, 2, 3, or 4 mechanisms; (d) Most common blocking sequence.

|  | **(i) Medium ground finches** | **(ii) Cactus  finches** | **(iii) Small ground finches** | **(iv) Woodpecker finches** |
| --- | --- | --- | --- | --- |
| **(a) Individuals who discovered 0, 1, 2, 3,  or 4 mechanisms** | 0 mech = - 1 mech = 3 ind (27%) 2 mech = 3 ind (27%) 3 mech = 2 ind (19%) 4 mech = 3 ind (27%) | 0 = - 1 = 2 (20%) 2 = 1 (10%) 3 = 2 (20%) 4 = 5 (50%) | 0 = 2 (22%) 1 = 6 (66%) 2 = 1 (11%) 3 = - 4 = - | 0 = - 1 = 1 (11%) 2 = 2 (22%) 3 = 4 (44%) 4 = 2 (22%) |
| **(b) Individuals who discovered 0, 1, 2, 3,  or 4 mechanisms in session 1** | 0 mech = 4 ind (36%) 1 mech = 5 ind (45%) 2 mech = 1 ind (9%) 3 mech = 1 ind (9%) 4 mech = - | 0 = 2 (20%) 1 = 6 (60%) 2 = 2 (20%) 3 = -  4 = - | 0 = 6 (67%) 1 = 3 (33%) 2 = - 3 = - 4 = - | 0 = 3 (33%) 1 = 6 (67%) 2 = - 3 = - 4 = - |
| **(c) Individuals who blocked 0, 1, 2, 3,  or 4 mechanisms** | 0 mech = 1 ind (9%) 1 mech = 3 ind (27%) 2 mech = 3 ind (27%) 3 mech = 3 ind (27%) 4 mech = 1 ind (9%) | 0 = - 1 = 2 (20%) 2 = 2 (20%) 3 = 1 (10%) 4 = 5 (50%) | 0 = 4 (44%) 1 = 5 (55%) 2 = - 3 = - 4 = - | 0 = - 1 = 1 (1%) 2 = 2 (22%) 3 = 4 (44%) 4 = 2 (22%) |
| **(d) Most common blocking sequence** | Toothpick Drawer Trap  Sliding | Toothpick Drawer Sliding  Trap | Toothpick - - - | Toothpick Drawer Trap  Sliding |
